# Lack of change in urate deposition by dual-energy computed tomography among clinically stable patients with long-standing tophaceous gout: a prospective longitudinal study

**DOI:** 10.1186/ar4343

**Published:** 2013-10-23

**Authors:** Ashwin Rajan, Opetaia Aati, Ramanamma Kalluru, Gregory D Gamble, Anne Horne, Anthony J Doyle, Fiona M McQueen, Nicola Dalbeth

**Affiliations:** 1 Bone and Joint Research Group, Department of Medicine, Faculty of Medical and Health Sciences, University of Auckland, 85 Park Road, Grafton, Auckland 1023, New Zealand; 2 Department of Anatomy with Radiology, Faculty of Medical and Health Sciences, University of Auckland, 85 Park Road, Grafton, Auckland 1023, New Zealand; 3 Department of Molecular Medicine, Faculty of Medical and Health Sciences, University of Auckland, 85 Park Road, Grafton, Auckland 1023, New Zealand

## Abstract

**Introduction:**

Dual-energy computed tomography (DECT) has potential for monitoring urate deposition in patients with gout. The aim of this prospective longitudinal study was to analyse measurement error of DECT urate volume measurement in clinically stable patients with tophaceous gout.

**Methods:**

Seventy-three patients with tophaceous gout on stable therapy attended study visits at baseline and twelve months. All patients had a comprehensive clinical assessment including serum urate testing and DECT scanning of both feet. Two readers analysed the DECT scans for the total urate volume in both feet. Analysis included inter-reader intraclass correlation coefficients (ICCs) and limits of agreement, and calculation of the smallest detectable change.

**Results:**

Mean (standard deviation) serum urate concentration over the study period was 0.38 (0.09) mmol/L. Urate-lowering therapy was prescribed in 70 (96%) patients. The median (interquartile range) baseline DECT urate volume was 0.49 (0.16, 2.18) cm^3^, and change in DECT urate volume was -0.01 (-0.40, 0.28) cm^3^. Inter-reader ICCs were 1.00 for baseline DECT volumes and 0.93 for change values. Inter-reader bias (standard deviation) for baseline volumes was -0.18 (0.63) cm^3^ and for change was -0.10 (0.93) cm^3^. The smallest detectable change was 0.91 cm^3^. There were 47 (64%) patients with baseline DECT urate volumes <0.91 cm^3^. Higher serum urate concentrations were observed in patients with increased DECT urate volumes above the smallest detectable change (*P* = 0.006). However, a relationship between changes in DECT urate volumes and serum urate concentrations was not observed in the entire group.

**Conclusions:**

In patients with tophaceous gout on stable conventional urate-lowering therapy the measurement error for DECT urate volume assessment is substantially greater than the median baseline DECT volume. Analysis of patients commencing or intensifying urate-lowering therapy should clarify the optimal use of DECT as a potential outcome measure in studies of chronic gout.

## Introduction

Dual-energy computed tomography (DECT) is a new advanced imaging method that allows rapid noninvasive assessment of urate deposition in patients with gout [1-3]. In a dual-source, dual-energy scan, two X-ray sources run simultaneously at two kilovolt levels (80 kV and 140 kV), with two corresponding detectors. These provide two spiral data sets that are acquired simultaneously in a single scan [4]. A specific display algorithm assigns different colours to materials of different chemical composition. This includes detection of the elementary chemical composition of urate, allowing visualisation of monosodium urate (MSU) crystal deposition. High sensitivity (78% to 100%), high specificity (79% to 100%) and intraobserver and interobserver reproducibility (intraclass correlation coefficients ≥0.95) of DECT urate volume measurements have been reported [1-3,5,6], suggesting that this tool may be useful for both diagnosis and monitoring of patients with gout. Several case series have described changes in DECT urate deposition in response to treatment with urate-lowering therapy [7,8]. However, a systematic analysis of the sensitivity to change and measurement error of DECT has not been published. This information is essential in determining the role of DECT as a valid outcome measurement modality for use in clinical studies of chronic gout [9,10]. The primary aim of this prospective, longitudinal study was to analyse measurement error of DECT in the assessment of urate deposition in clinically stable patients with tophaceous gout. We also wished to examine the relationships between DECT volumes and clinical features of gout, as well as the correlations between changes in these measures.

## Methods

Seventy-three patients with tophaceous gout on stable therapy were prospectively recruited between June 2010 and August 2012 from rheumatology outpatient clinics in Auckland, New Zealand. All patients had a history of gout according to the 1977 American Rheumatism Association classification criteria [11] and at least one subcutaneous tophus found during clinical examination. Women of childbearing age were excluded from the study. The Northern Regional Ethics Committee approved this study. All patients provided written informed consent before inclusion into the study.

The patients attended two study visits: at baseline and at 12 months. Clinical assessment was undertaken as part of each study visit. Clinical data recorded included demographic details (age, sex and ethnicity), gout history (disease duration, frequency of gout flares and gout treatments), medications, physical examination (including tophus count, swollen joint count and tender joint count), Health Assessment Questionnaire II (HAQ-II) score, and laboratory tests, including serum urate concentration and C-reactive protein. Urate-lowering therapy was managed by the patients’ usual clinician and no alterations were made to urate-lowering therapy as part of the study protocol. Urate-lowering therapies available at the time of the study were allopurinol and probenecid. All serum urate concentrations measured as part of clinical care over the 12-month period were also recorded. The median and interquartile range (IQR) of all serum urate tests obtained over the study period was four tests [3,5]. There was a high correlation between mean serum urate concentrations for the two study visits (baseline and year 1) and the mean serum urate concentrations using all measurements over the entire study period (*r* = 0.95, *P* < 0.0001). Therefore, to preserve uniformity of the data for all participants, the serum urate measurements obtained at the baseline and year 1 study visits were analysed.

DECT scans of the feet were performed on the day of each study visit on a dual-source X-ray tube 128 detector row scanner (SOMATOM Definition Flash; Siemens Healthcare, Erlangen, Germany). The patients were positioned feet first in a supine position with the feet in a plantar-flexed position. The scan was acquired in a craniocaudal direction, starting proximally 5 cm from the ankle joint to the toe tips. Both ankles and feet were scanned axially in one helical image acquisition. The gantry rotation time was 1 second, and the field of view (reconstruction diameter) was 30 cm. All scans were performed using the same image protocol: acquisition at 128 mm × 0.6 mm and pitch of 0.7. X-ray tube 1 was operated at 80 kV/260 mA, and tube 2 was operated at 140 kV/130 mA. The images were reconstructed on a bone algorithm, 512 × 512 matrix, to 0.75-mm slices with a 0.5-mm increment. Additional reconstructions were done using a soft-tissue algorithm and 512 × 512 matrix, also to a 0.75-mm slice with a 0.5-mm increment. The images were viewed as 0.75-mm slices on a picture archiving communication system.

Two readers with training and experience in automated DECT volume assessments (a rheumatologist, RK, and a rheumatology research assistant, OA) [12] analysed the DECT scans (at baseline and at 12 months) for the total volume of urate deposition in both feet. Paired scans were read in chronological order to increase the sensitivity of the analysis [13]. The readers were blinded to each other’s measurements and all clinical measures, including serum urate concentrations. A proprietary workstation (MultiModality WorkPlace, Siemens Healthcare) was used with proprietary software (*syngo* MMWP #MM; Siemens Healthcare). For the 80-kV images, fluid was set at 50 Hounsfield units (HU), the ratio for urate at 1.28, minimum HU at 150 and smoothing range of 5. For the 140-kV images, fluid was set at 50 HU and maximum HU at 500. Initial optimisation work showed that the ratio used in this study was associated with minimal artefact and that manual removal of apparent artefact led to no difference in the volumes measured (*n* = 25, mean (SD) volume without apparent artefact removed 4.064 (6.775) and with apparent artefact removed 4.055 (6.864); *P* = 0.76). Therefore, for the subsequent analysis, the region of volume measurement (both feet) was manually outlined by each reader, with no attempts to manually remove apparent artefacts within the region of interest.

Radiation dose estimates and risk analysis were performed by an experienced medical physicist in collaboration with the National Radiation Laboratory of New Zealand. The dose estimates were calculated from the exposure factors used for computed tomography (CT) scans of the feet. The effective dose was then calculated based on the methods from International Commission on Radiological Protection Publication 60 [14]. For foot and ankle CT, a slice 1-cm thick gave an effective dose of 0.0018 mSv using PCMXC software (Radiation and Nuclear Safety Authority, Helsinki, Finland). The range of 30 cm gave a dose of 0.054 mSv.

Data were analysed using SPSS statistical software (SPSS, Inc, Chicago, IL, USA) and GraphPad Prism v5 software (La Jolla, CA, USA). Medians with IQR and percentages were used to describe the clinical characteristics of patients. Parametric and nonparametric *t*-tests and χ^2^ analysis were used to determine the differences between groups. In the case of three groups, one-way analysis of variance (ANOVA) with Tukey’s *post hoc* test was used. Interobserver reproducibility was assessed by intraclass correlation coefficient (ICC) and limits of agreement by Bland-Altman analysis. The smallest detectable change (SDC) and smallest detectable difference were calculated as described by Bruynesteyn *et al*. [14]. Spearman’s rank correlations were used to determine the relationships between clinical features and DECT measurements. All tests were two-tailed, and *P* < 0.05 was considered statistically significant.

## Results

### Clinical characteristics

The clinical characteristics of the patients at baseline and after one year are shown in Table 1. Patients were predominantly middle-aged men, and more than half were of Māori or Pacific ethnicity. The median disease duration was more than 20 years. Urate-lowering therapy was prescribed in 70 patients (96%), most commonly allopurinol in (62 patients; 85%). No patients were taking febuxostat or pegloticase. The clinical features of gout did not change over the one-year observation period. For serum urate tests conducted at baseline and year 1, the mean (SD) value was 0.38 mmol/L (0.09). For all serum urate tests over the course of the study period, the mean (SD) value was 0.39 (0.09) (*P* = 0.52 compared with serum urate concentrations at the two study visits). Serum urate concentrations below target (less than 0.36 mmol/L) were present in 34 patients (47%) at baseline and in 29 patients (40%) after one year. There were 21 patients (29%) with sodium urate levels less than 0.36 mmol/L at both time points.

**Table 1:** **Clinical characteristics of the patients at baseline and at year 1**^**a**^

**Characteristics**	**Baseline**	**Year 1**
Male sex, *n* (%)	67 (92%)	**–**
Age, years	58 (49 to 65)	**–**
Pacific ethnicity, *n* (%)	25 (34%)	**–**
Māori ethnicity, *n* (%)	18 (25%)	**–**
European and other ethnicities, *n* (%)	30 (41%)	**–**
Gout disease duration, years	23 (16 to 28)	**–**
Flare frequency (in the preceding three months)	0 (0 to 2)	0 (0 to 2)
Allopurinol use, *n* (%)	62 (85%)	62 (85%)
Allopurinol dose (*n* = 62), mg/day	300 (200 to 300)	300 (200 to 300)
Colchicine use, *n* (%)	33 (45%)	32 (44%)
Nonsteroidal anti-inflammatory drug use, *n* (%)	46 (63%)	44 (60%)
Prednisone use, *n* (%)	11 (15%)	12 (16%)
Health assessment questionnaire II score	0.2 (0 to 0.55)	0.15 (0 to 0.58)
Swollen joint count	0 (0 to 0)	0 (0 to 0)
Tender joint count	0 (0 to 1)	0 (0 to 1)
Number of subcutaneous tophi	3 (2 to 9)	3.5 (2 to 8)
Serum creatinine, μmol/L	99 (84 to 112.5)	96 (81 to 112)
C-reactive protein, mg/L	2.4 (1 to 5)	2.1 (1 to 6)
Serum urate, mmol/L	0.36 (0.18 to 0.46)	0.37 (0.32 to 0.46)
Serum urate <0.36 mmol/L, *n* (%)	34 (47%)	29 (40%)
Serum urate <0.36 mmol/L at both time points, *n* (%)	21 (29%)	

^a^Unless otherwise specified, data are presented as medians (IQRs).

### Dual-energy computed tomography urate volumes at baseline and follow-up

Volumes and change values for each reader are shown in Table 2. All patients had urate deposition detected by DECT at baseline. The median (IQR) baseline DECT urate volume (mean for reader 1 and reader 2) was 0.49 cm^3^ (0.16 to 2.18). The median (IQR) follow-up DECT urate volume was 0.51 cm^3^ (0.18 to 2.08). The median (IQR) change in DECT urate volume was -0.01 cm^3^ (-0.40 to 0.28), and the mean (SD) change was -0.63 cm^3^ (2.49).

**Table 2:** Scoring results for each reader and interreader reproducibility analysis for dual-energy computed tomography urate volume and volume change measurements

**Parameter**	**Volume at baseline**	**Volume at year 1**	**Change in volume at year 1**
**Reader 1, median (IQR)**	0.59 cm^3^ (0.14 to 2.3)	0.60 cm^3^ (0.21 to 2.2)	0.00 cm^3^ (-0.44 to 0.48)
**Reader 1, mean (SD)**	2.9 cm^3^ (7.1)	2.3 cm^3^ (5.2)	-0.58 cm^3^ (2.6)
**Reader 2, median (IQR)**	0.42 cm^3^ (0.13 to 2.1)	0.29 cm^3^ (0.12 to 1.8)	-0.03 cm^3^ (-0.37 to 0.07)
**Reader 2, mean (SD)**	2.8 cm^3^ (6.9)	2.1 cm^3^ (5.2)	-0.68 cm^3^ (2.5)
**Average (reader 1 and reader 2), median (IQR)**	0.49 cm^3^ (0.16 to 2.2)	0.51 cm^3^ (0.18 to 2.1)	-0.01 cm^3^ (-0.40 to 0.28)
**Interreader intraclass correlation coefficient (95% CI)**	1.0 (0.99 to 1.0)	0.99 (0.99 to 1.0)	0.93 (0.89 to 0.96)
**Interreader bias (SD)**	-0.18 cm^3^ (0.63)	-0.27 cm^3^ (0.62)	-0.10 cm^3^ (0.93)
**Smallest detectable difference, average measurement (%)**	44.1%	55.2%	289%
**Smallest detectable change**	–	–	0.91 cm^3^

### Reproducibility of dual-energy computed tomography urate measurements: total volume and change analysis

**Figure 1:**
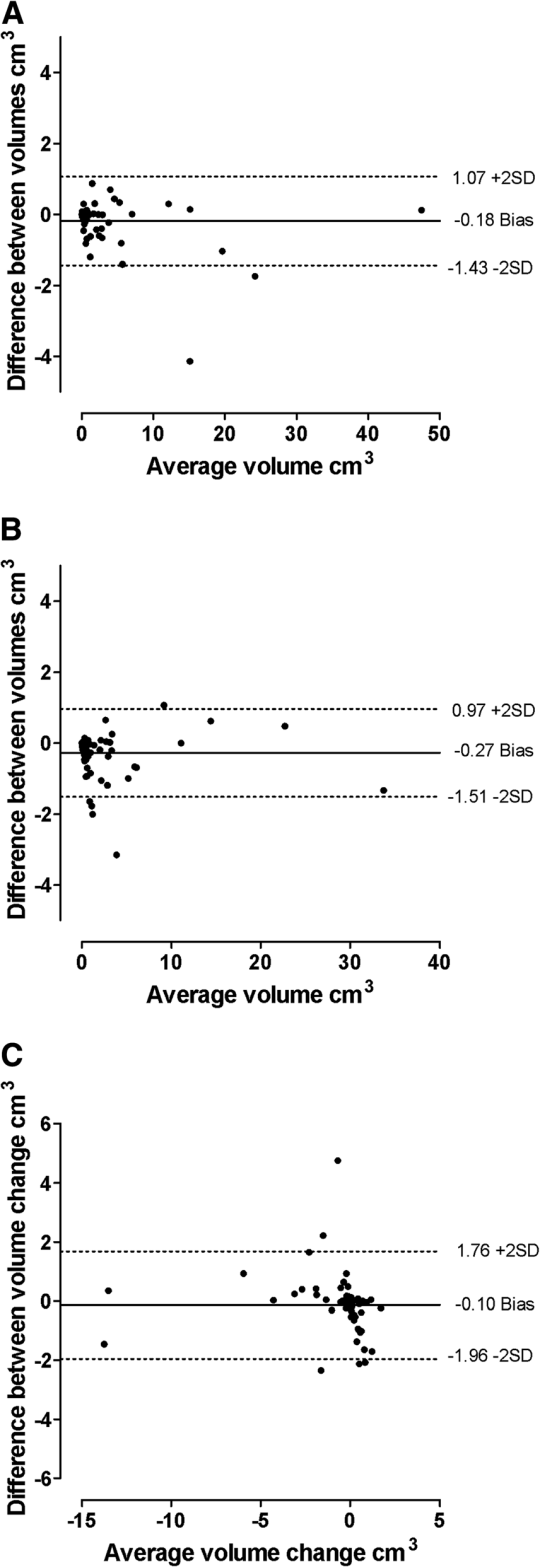
**Bland-Altman plots for interobserver reproducibility analysis. (A)** Dual-energy computed tomography (DECT) urate volume at baseline. **(B)** DECT urate volume at year 1. **(C)** Change in DECT urate volume over the course of one year. Solid line shows bias, and dashed lines show the limits of agreement.

The interobserver reproducibility analysis is shown in Table 2. Intraclass correlation coefficients for both volume assessment and change values were excellent (greater than 0.92). Bias was low; however, limits of agreement for the change values were considerably larger than the observed changes. Bland-Altman plots illustrating the interobserver limits of agreement for volume and volume change measurements are shown in Figure 1. Inspection of the plots shows no compelling evidence for differing bias with increasing average volume or average volume change. The SDC was calculated to be 0.91 cm^3^. There were 47 patients (64%) with baseline DECT urate volumes less than 0.91cm^3^.

### Relationship between clinical features and dual-energy computed tomography urate volume at baseline

Baseline DECT urate volume correlated with the baseline subcutaneous tophus count (*r* = 0.62, *P* < 0.0001), C-reactive protein (*r* = 0.36, *P* = 0.002), swollen joint count (*r* = 0.32, *P* = 0.007) and flare frequency (*r* = 0.26, *P* =0.03). There was no correlation between baseline DECT urate volume and other clinical variables at the baseline study visit, such as tender joint count (*r* = 0.07, *P* = 0.56) and HAQ-II score (*r* = 0.06, *P* = 0.61). There was a trend towards a positive correlation with the serum urate concentration (*r* = 0.22, *P* = 0.06).

### Relationship between change in dual-energy computed tomography urate volume and mean serum urate concentration

**Figure 2:**
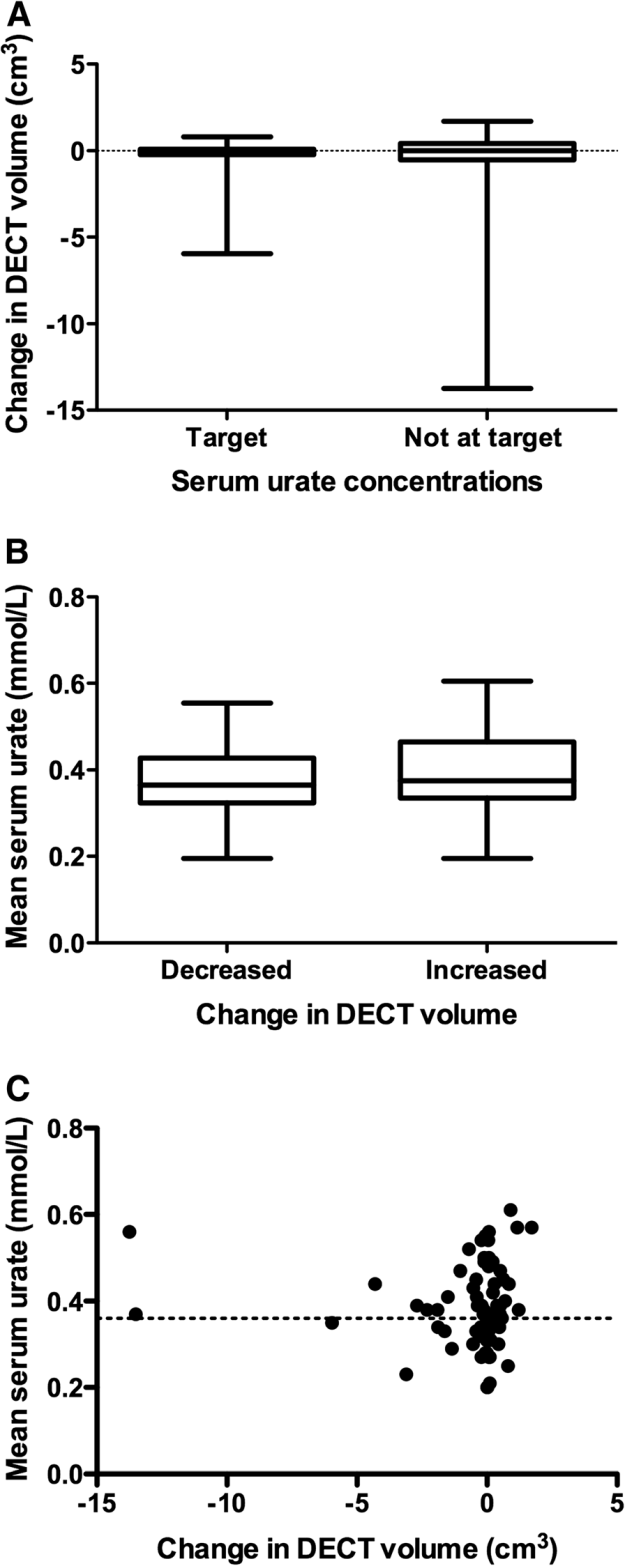
**Relationship between changes in dual-energy computed tomography urate volumes and serum urate concentrations. (A)** Box-and-whisker plot showing dual-energy computed tomography (DECT) urate volume in patients with serum urate concentrations at the therapeutic target (less than 0.36 mmol/L) at both time points and those not at target at both time points. **(B)** Box-and-whisker plot showing mean serum urate concentrations (baseline and year 1) in patients with decreased and increased DECT urate volumes over the one-year observation period. **(C)** Scatterplot showing the relationship between change in DECT urate volume from baseline to year 1 and mean serum urate concentrations (baseline and year 1).

**Figure 3:**
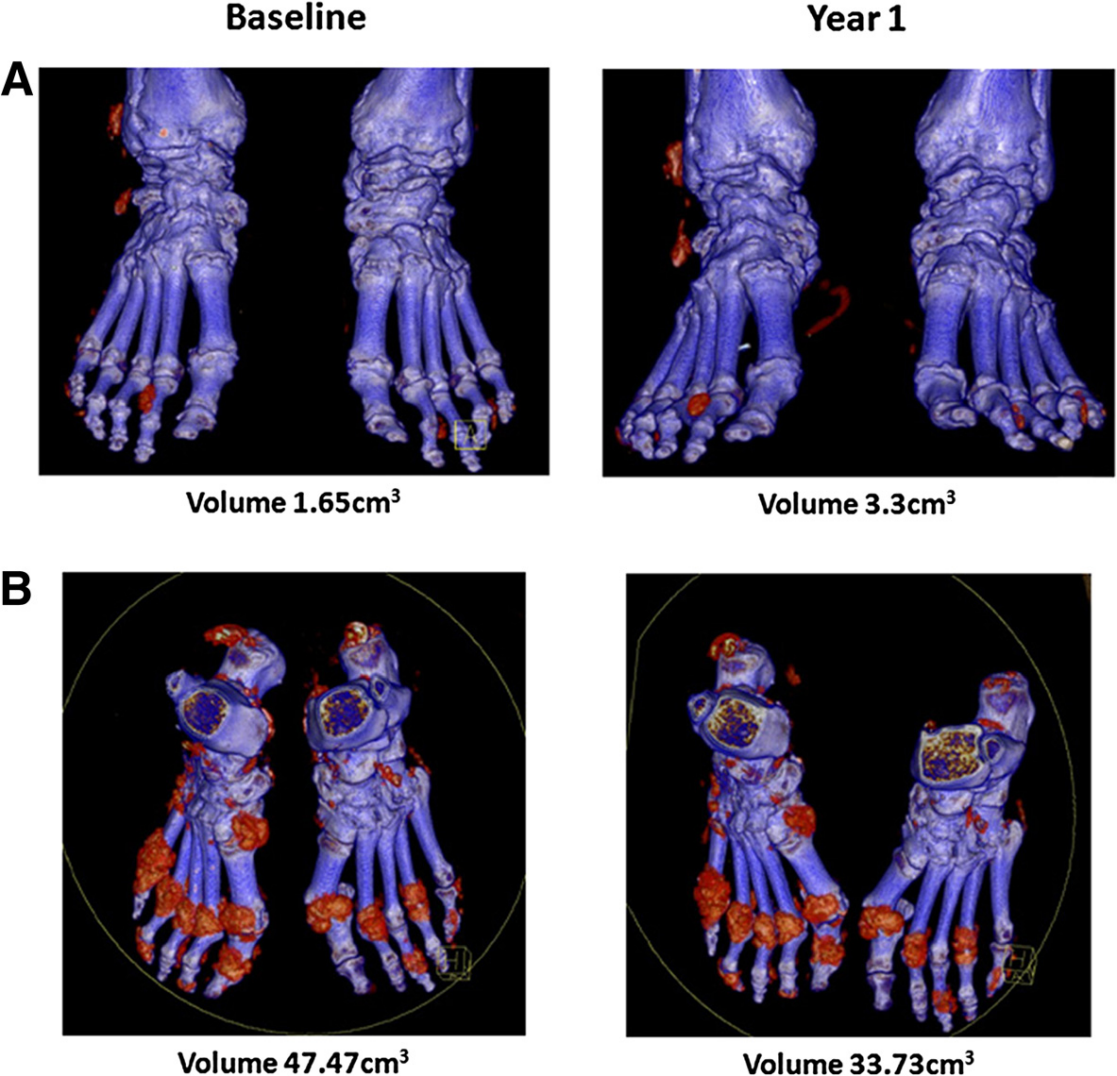
**Examples showing lack of association between changes in serum urate concentration and changes in dual-energy computed tomography urate volume.** Paired dual-energy computed tomography (DECT) scans from two patients with gout with hyperuricaemia throughout the study period. **(A)** Paired scans from a patient with 0.49 mmol/L serum urate concentration at baseline visit, and 0.57 mmol/L at year 1 visit. DECT urate volume increased from 1.65cm^3^ to 3.3 cm^3^. **(B)** Paired scans from a patient with a 0.55 mmol/L serum urate concentration at baseline and 0.56 mmol/L at year 1. DECT urate volume decreased from 47.47 cm^3^ to 33.73 cm^3^.

Given the well-documented relationship between serum urate concentration and other clinical outcomes in gout, we hypothesized that patients with serum urate concentrations below the therapeutic target (less than 0.36 mmol/L) throughout the study period would have greater reductions in urate volume as assessed by DECT. When the patients were separated into those who had serum urate concentrations at target at both time points and those who did not, however, there was no difference in the change in DECT urate volume (*P* = 0.79) (Figure 2A). Similarly, when the patients were separated into those with increased DECT urate volumes and those with reduced DECT urate volumes, there was no difference in the mean serum urate concentration between these groups over the study period (*P* = 0.34) (Figure 2B). Analysis of year 1 serum urate concentrations only and analysis of all serum urate concentrations obtained throughout the one-year period did not alter the findings of this analysis (*P* = 0.34 and *P* = 0.17, respectively; data not shown). The changes in DECT urate volume did not correlate with serum urate concentrations at baseline, serum urate concentrations at year 1 or changes in serum urate concentration or mean serum urate concentration over the one-year period (*r* < 0.14, *P* > 0.28 for all) (Figure 2C). Examples of the variable results in individual patients are shown in Figure 3.

**Figure 4:**
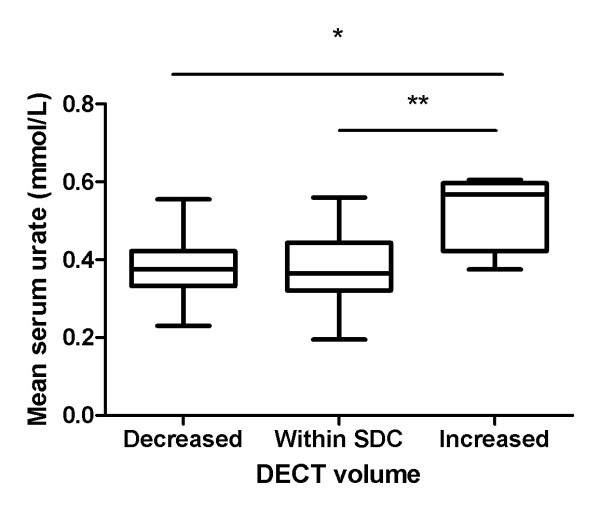
**Relationship between changes in dual-energy computed tomography urate volume and mean serum urate concentration with analysis using the smallest detectable change as the measurement error.** Box-and-whisker plot showing mean serum urate concentrations (baseline and year 1) in patients with decreased dual-energy computed tomography (DECT) urate volumes below the smallest detectable change (SDC), changes in DECT urate volume within the SDC and increased DECT urate volume above the SDC. *P < 0.05, **P < 0.01 by Tukey’s *post hoc* test.

Considering the SDC of 0.91 cm^3^ as the measurement error, we next analysed the mean serum urate concentrations in those patients with increased volume above the SDC, changes within the SDC and reduced volume below the SDC. This analysis demonstrated higher mean serum urate concentrations in the group with increased DECT urate volume above the SDC (*P* = 0.006 by one-way ANOVA) compared with both changes within the SDC and reductions below the SDC (*P* < 0.01 and *P* < 0.05, respectively) (Figure 4). In this group, the change in DECT urate score correlated with year 1 serum urate concentrations (0.39; *P* = 0.048), with a similar trend observed for the mean serum urate concentration over the one-year period (*r* = 0.385, *P* = 0.052).

### Relationship between change in other clinical features and change in dual-energy computed tomography urate volume over one year

In the entire group, the change in DECT urate volume correlated with the change in three-month flare frequency (*r* = 0.27, *P* = 0.02). However, the change in DECT urate volume did not correlate with changes in other clinical features of gout, such as subcutaneous tophus count, C-reactive protein, HAQ-II score, tender joint count or swollen joint count (*r* < 0.20, *P* > 0.09 for all). Similarly, for the group in which there were changes in DECT urate volume above the SDC, the change in DECT urate score correlated with changes in the frequency of gout flares (*r* = 0.41, *P* = 0.037), but not with changes in subcutaneous tophus count, C-reactive protein, HAQ-II score, tender joint count or swollen joint count (*r* < 0.29, *P* > 0.15 for all).

## Discussion

The aim of this study was to examine measurement error of DECT urate volumes in patients with clinically stable tophaceous gout. The group of patients in this analysis were on stable therapy, and, overall, we did not observe differences in the clinical features of gout or serum urate concentrations during the one-year period. Consistent with these observations, the median DECT urate volume did not change over the one-year period in the group overall. As previously reported by our group and others [2,5], the current study confirms high interreader agreement in DECT assessment of urate volume with very high ICCs and low limits of agreement. Although low bias was observed, however, the measurement error was substantially greater than the observed change in this clinically stable group. A key finding of this analysis was the SDC of 0.91 cm^3^. In this study, we recruited patients with established, long-standing gout, and all participants had at least one subcutaneous tophus. However, almost two-thirds of these patients had DECT urate volumes in the feet that were lower than the SDC. Together, these observations indicate that even complete resolution of urate deposition in the majority of our patients would have been within the measurement error. This observation has important implications for the design of clinical studies using DECT as an outcome measure. On the basis of the data available, it is possible that DECT will not have sufficient sensitivity in studies of patients with less advanced gout or without clinically apparent tophi. Scanning additional sites (for example, the location of the clinically apparent tophus) may improve the accuracy of this method. However, the sensitivity of DECT in this manner, compared with simple clinical measurement or other imaging techniques, requires careful evaluation.

MSU crystal dissolution occurs in the presence of persistent subsaturation urate concentrations [15]. Several long-term observational studies have indicated that clinical benefit occurs in people with gout in the context of long-term serum urate-lowering below 0.36 mmol/L [16-18]. For this reason, we analysed the change in DECT urate volumes based on the presence of target serum urate concentrations over the course of the study period. Higher mean serum urate concentrations were observed in patients with increased DECT urate volumes above the measurement error. However, we did not observe a consistent relationship between changes in DECT urate volumes and serum urate concentrations. A number of features of this study may have contributed to the lack of anticipated change based on the serum urate concentrations. Most importantly, the patients included in this study had long-standing clinically stable disease; therefore, changes in urate deposition, particularly without profound reductions in serum urate concentrations, may not have occurred over the course of just one year. Additionally, the degree of serum urate lowering in those with serum urate concentrations below target may not have been sufficiently intense to result in corresponding reductions in DECT urate volume. It is worth noting that the studies of febuxostat and allopurinol did not identify a significant reduction in physical measurements of subcutaneous tophus size over a one-year period in patients with serum urate concentrations below 0.36 mmol/L [19]. Our findings differ from those of a 12-month study which determined sensitivity to changes detected by ultrasonography (US) in the assessment of tophus size in 14 patients with gout [20]. The US study demonstrated a strong relationship between tophus size and average serum urate concentrations during the study period. In contrast to our study of clinically stable patients, however, all patients in the US study commenced urate-lowering therapy at the start of the study, with median serum urate concentrations of 0.54 mmol/L at baseline and 0.34 mmol/L at follow-up. It should also be noted that US captures both urate volume and the tissue response within the tophus, whereas DECT captures only the urate volume, which can be highly variable within the tophus [5].

DECT is an emerging technology, and current hardware and software may not be optimal for the purposes of long-term monitoring of urate deposits in gout. Artefacts can occur using this technique, and small variations in the threshold settings can substantially alter the urate signal [21,22]. Importantly, all patients were scanned using the same scanner and an identical acquisition protocol (including the same orientation and positioning), and all analyses were performed using the same settings. The ratio of 1.28 was used for all scoring in this study. Our recent data suggest that this setting may have lower sensitivity for detecting urate deposits using DECT [22], but this setting is also associated with minimal artefact, as confirmed in our initial optimisation work. It is uncertain whether protocols with higher ratios and manual removal of artefact have less measurement error. Further work to optimize the methodology may reduce the measurement error.

## Conclusions

In this prospective longitudinal study of patients with tophaceous gout on stable conventional urate-lowering therapy, we have shown that the measurement error for DECT urate volume assessment is substantially greater than the median baseline DECT volume. Analysis of patients commencing or intensifying urate-lowering therapy, using modified protocols which include sites of clinically apparent tophi, should clarify the optimal use of DECT as a potential outcome measure in studies of chronic gout.

## Abbreviations

ANOVA: Analysis of variance; CI: Confidence interval; DECT: Dual-energy computed tomography; HAQ-II: Health assessment questionnaire II; HU: Hounsfield units; ICC: Intraclass correlation coefficients; IQR: Interquartile range; MSU: Monosodium urate; SD: Standard deviation; SDC: Smallest detectable change; SDD: Smallest detectable difference; SU: Serum urate; US: Ultrasonography.

## Competing interests

The authors have no conflicts of interest to declare.

## Authors’ contributions

AR coordinated the analysis, assisted with data entry and drafted the manuscript. OA recruited patients, analysed the DECT scans and managed the data entry. RK contributed to study design and analysed the DECT scans. GDG assisted with data analysis and interpretation. AH contributed to patient recruitment and data management. AJD and FMM contributed to study design, data interpretation and manuscript drafting. ND (the guarantor) accepts full responsibility for the work and the conduct of the study, had access to the data and controlled the decision to publish. ND conceived of the study, contributed to the data interpretation and drafted the manuscript. All authors read and approved the final manuscript.
